# Happiness and Depression in Adolescence after Maternal Smoking during Pregnancy: Birth Cohort Study

**DOI:** 10.1371/journal.pone.0080370

**Published:** 2013-11-12

**Authors:** Ana Maria Baptista Menezes, Joseph Murray, Mitzi László, Fernando C. Wehrmeister, Pedro C. Hallal, Helen Gonçalves, Maria Cecilia F. Assunção, Carolina Baptista Menezes, Fernando C. Barros

**Affiliations:** 1 Post-Graduate Program in Epidemiology, Federal University of Pelotas, Pelotas, Rio Grande do Sul, Brazil; 2 Department of Psychiatry, Cambridge, United Kingdom; 3 Department of Psychology, Federal University of Pelotas, Pelotas, Rio Grande do Sul, Brazil; 4 Post-Graduate Program in Health and Behavior, Catholic University of Pelotas, Pelotas, Rio Grande do Sul, Brazil; University of Oxford, United Kingdom

## Abstract

**Background:**

Prenatal cigarette smoke exposure may have adverse psychological effects on offspring. The objective was to assess the association between parental smoking during pregnancy and offspring happiness at age 18, as well as depression.

**Methodology:**

Participants were part of a birth cohort study in Pelotas, Brazil (5,249 participants). Happiness was measured by the *Subjective* *Happiness* *Scale*, a Likert-like scale with four questions generating a score from 1 to 7, with ≥ 6 indicating "happiness". Depression was measured using the Mini International Psychiatric Interview.

**​Results:**

About one third of mothers reported having smoked during pregnancy and 4.6% reported smoking 20 or more cigarettes a day. The prevalence of happiness was 32.2% (95% CI 30.8; 33.7), depression 6.8% (95% CI 6.1; 7.6), and simultaneous happiness and depression less than 1%. The prevalence of offspring happiness decreased as smoking in pregnancy increased, even after control for confounding variables, showing an OR = 0.79 [95% CI 0.55; 1.13]. The opposite happened to depression; the prevalence of offspring depression increased as smoking in pregnancy increased (<20 cigarettes/day OR = 1.38 [95% CI 1.03; 1.84] and ≥20 cigarettes/day OR = 2.11[95% CI 1.31; 3.40]. Smoking by the partner was associated with decreased offspring happiness after adjustment for confounders, but did no show association with offspring depression.

**Conclusions:**

Offspring were less likely to be happy and more likely to be depressed if their mother smoked during pregnancy, and less likely to be happy if their father smoked during mother’s pregnancy. Although we can not affirm that this is a “causal pathway”, public policies to reduce smoking in pregnancy could improve the health of the offspring in the short and long term.

## Introduction

There is evidence that prenatal cigarette smoke exposure can have short- and long-term adverse psychological effects on offspring, both in human [1,2] and animal studies [3]. Previous studies have tended to focus on the association between cigarette smoke exposure during pregnancy and externalizing and internalizing behavioural problems. Externalizing behavioural problems, such as aggression, attention deficits and substance use in children have all been linked to prenatal exposure to tobacco smoke [4,5]. It has been demonstrated that in adults aged 22, externalizing and internalizing problems are associated with prenatal exposure to tobacco, even after controlling for potential confounders, such as parental mental health, alcohol and marijuana exposure, and socio-demographic variables [1]. However, this association is not consistent across ages. Other longitudinal assessments of offspring at ages 4 [6], 5 [7], 10 [8], 14 [9], and 11-13-16 [10] failed to detect associations with internalizing problems, even though many showed an association between tobacco exposure and externalizing and attention problems [6,7,9].

Recently, there has been a focus on the study of happiness as a separate phenomenon to depression and internalizing problems. Happiness is conceptualised as an important outcome in its own right, distinct from the absence of depressive disorder, possibly with its own specific causal mechanisms [11]. It has been demonstrated that lower levels of happiness and well-being are associated with increased mortality [12] and greater risk of chronic illnesses [13]. Given the social and economic impact of poor mental health worldwide, and growing recognition of the importance of happiness or “well being”, the value of prevention and early intervention is highly emphasised [14]. We found no studies that have assessed the association between prenatal exposure to tobacco and happiness. Smoking during pregnancy could be one possible preventable risk factor for the development of maladaptive emotional functioning among offspring. 

There is no consensus in the literature on the underlying biological pathway involved in the association of maternal smoking during pregnancy and some outcomes in children or adolescents. As has been pointed out by several authors [15–18], one of the difficulties for the interpretation of results from several studies is to disentangle prenatal exposures effects from environmental and genetic factors. Animal studies suggest a direct effect of maternal smoking during pregnancy and mood changes through a decrease in certain neurotransmitters as a result of prenatal nicotine exposure. 

Using a birth cohort design, the primary aim of this study was to assess the association between cigarette smoke exposure during pregnancy with happiness of the teenager at age 18, taking into account the role of confounding and mediating factors. Importantly, unhappy people were not considered necessarily depressed, and happiness and depression were analysed as two separate outcomes. Depression is associated with internalizing behavioral problems and can be a very disabling condition, associated with many negative outcomes, including impaired social, educational, occupational, and familial functioning [19].

## Methods

In the calendar year of 1993, all deliveries taking place in the four hospitals of the city of Pelotas, Brazil were monitored. All mothers who lived in the urban area of the city at that time were invited to participate in a research project, in which newborns would be followed up from birth to adult life. All but 16 mothers agreed to take part, resulting in a total cohort size of 5,249 individuals. Within 24 hours of delivery, mothers answered a questionnaire and newborns underwent a series of anthropometric measurements. The questionnaire collected information on maternal smoking during pregnancy (no, <20 cigarettes per day, ≥20 cigarettes per day), partner smoking (no, yes), family income (divided into quintiles for analyses), whether or not the pregnancy was planned, maternal perception on father’s support during gestation (low, intermediate, high), maternal alcohol consumption during pregnancy (no, yes), and type of delivery (vaginal, C-section). 

At 11, 15 and 18 years of age, all cohort members were sought for follow up visits. Out of the 5,249 participants, we were able to locate 87.5% at 11 years, 85.7% at 15 years and 81.4% at 18 years of age. At the 11 years follow up visit, mothers answered the Self Report Questionnaire (SRQ) [20]. We used a cut-off point of eight points to classify women as normal or presenting minor psychiatric disorders within the previous 30-day period. At the 15 years visit, we administered the Strengths and Difficulties Questionnaire (SDQ) [21]. This was answered by mothers and used to judge the adolescent’s mental health in the 6 months before the interview; it is aimed at measuring conduct problems, emotional problems and symptoms of inattention and hyperactivity. These were classified as normal (0-13 points), borderline (14-16 points) or abnormal (17-40 points).

At the 18 years visit, we administered the Subjective Happiness Scale (SHS) [22] for measuring happiness (no time frame) and the Mini-International Neuropsychiatric Interview (MINI) for measuring depression within a period of the “last 15 days” [23] to all participants of the cohort. The SHS scale comprises four questions with answers ranging from 1 to 7, as follows:

1. In general, I consider myself

  1 (not a very happy person) 2 3 4 5 6 7 (a very happy person)

2. Compared to most of my peers, I consider myself:

  1 (less happy) 2 3 4 5 6 7 (a very happy person)

3. Some people are generally very happy. They enjoy life regardless of what is going on, getting the most out of everything. To what extent does this characterization describes you?

  1 (not at all) 2 3 4 5 6 7 (a great deal)

4. Some people are generally not very happy. Although they are not depressed, they never seem as happy as they might be. To what extent does this describe you?

  1 (not at all) 2 3 4 5 6 7 (a great deal)

For the first three questions, a score of 7 indicates the ‘happiest’ status. Answers to the last question were reversed to indicate the same. Therefore, the total score ranges from 4 to 28 points. We then calculated the average score for each participant by simply dividing the total score by four. For analyses we considered those who obtained a mean value of six or higher in SHS scale as happy and others as normal-unhappy [24]. The Cronbach's alpha statistics measured in our own sample was 0.61.

Regarding the validity of the instruments used in this paper it should be mentioned that the validity of the SHS was compared to other similar tests such as Satisfaction with Life Scale and the Positive Affect Scale, showing a positive Pearson correlation of 0.66 and a moderate correlation of 0.49, respectively [25]. The SRQ tool is used to measure basic anxiety and depression and it has been validated in a Brazilian sample of 485 subjects with a mean age of 40,04 (SD=15,89) [26]; it was compared with the DSM-IV-TR as the gold standard showing a 86.3% of sensitivity, 89.3% of specificity, a discriminant power of 0.9 for psychiatric screening and a 0.86 Cronbach’s alpha. The SDQ instrument has been validated in Brazil [27] and compared with a diagnostic instrument (Development and Well-Being Assessment) in the Pelotas birth cohort at 11 years old [28] showing a sensitivity of 78.2%, specificity of 70.4%, positive predictive value of 48.2%, negative predictive value of 90.2% and an area under the curve of 74.0%. The MINI DSM-IV represents an economic option for identifying diagnostic cases according to international criteria, in clinical trials and epidemiological studies; it is available in almost 30 languages, including a Brazilian version [29]. The depression component of this instrument has been tested against CID and SCID-P showing the following results, respectively: 0.73 and 0.84 for kappa statistics, 0.94 and 0.96 for sensibility, 0.79 and 0.88 for specificity, 0.82 and 0.87 for predictive positive value and 0.93 and 0.97 for predictive negative value.

Three teenagers fulfilled in exclusion criteria due to mental disability (they were not eligible for the MINI interview according to the general manual of procedures of the Pelotas Birth Cohort); we also had some missing information for the MINI questionnaire for another 50 teenagers who were excluded from analyses. 

Data analyses included a description of the sample in terms of perinatal and adolescent variables. For the multivariate analysis we used logistic regression. The main exposure variables were maternal and partner smoking during pregnancy. We ran three different models: unadjusted, adjusted for confounders only (sex, family income, planned pregnancy, father support during pregnancy, maternal alcohol consumption, type of delivery and maternal SRQ when adolescents were 11 years of age), and adjusted for confounders and mediator (all variables mentioned before plus adolescent SDQ at the age of 15 years). A mediator analysis was carried out using the g-computation formula [30] showing that from the total effect of smoking intra utero and depression or happiness more than 75% was explained by the direct effect and not by the mediator. Although the mediator effect was small, we opted for maintaining this variable as a mediator in the model.

Calculations of prevalence of happiness with 95% confidence intervals were carried out using the SHS test when the teenager was 18 and the same calculations of prevalence of depression using the MINI test were performed.

The study protocol was approved by the Federal University of Pelotas Ethics Committee. Cohort members and their mothers provided written informed consent prior to each interview. 

## Results

Of the 5,249 cohort members, we were able to locate 4,106 participants at 18 years of age; 49.1% of them were men. The response rate was 81.4%, including those who died before reaching 18 (n=169). 

From the total sample, 4,053 individuals had information for happiness and depression; 2,510 (61.9%) displayed neither happiness nor depression, 1,266 (31.3%) presented happiness, and 251 (6.2%) were depressed only. Of those not depressed (N = 26), 0.6% were classified as happy, confirming that happiness is not the same as absence of depression.

Table 1 shows the distribution of the exposure, confounding and mediating variables. More than half of the mothers reported that the pregnancy was not planned. The proportion of mothers reporting high support from the father during pregnancy was 88%. Only 5.2% reported consuming alcohol during pregnancy. The proportion of C-sections was 31%. At the 11 years follow up visit, 31% of the mothers were classified as presenting minor psychiatric disorders. At 15 years of age, 27% of the adolescents were classified as abnormal in the SDQ questionnaire. In relation to the main exposure variables, 33% of the mothers reported smoking during pregnancy; only 4.6% reported smoking 20 or more cigarettes per day. The prevalence of partner smoking was 49.5% 

**Table 1:** Sample distribution at 18 years old according to perinatal characteristics and prevalence of happiness and depression.

**Variable**	**N (%)**	**Happiness (% and 95% CI)N=4106**	**Depression (% and 95% CI)N=4053**
***Sex***		*p=0.081*	*p<0.001*
Male	2015 (49.1)	30.9 (28.9; 33.0)	3.5 (2.7; 4.3)
Female	2091 (50.9)	33.5 (31.5; 35.5)	10.0 (8.7; 11.3)
***Family income (quintiles)***		*p<0.001*	*p<0.001*
1^st^ (lower)	781 (19.4)	26.2 (23.2; 29.3)	8.5 (6.6; 10.5)
2^nd^	943 (23.4)	31.1 (28.1; 34.0)	9.1 (7.3; 11.0)
3^rd^	702 (17.4)	30.3 (26.9; 33.7)	7.2 (5.3; 9.0)
4^th^	811 (20.1)	33.9 (30.6; 37.2)	4.3 (2.9; 5.7)
5^th^ (higher)	797 (19.8)	39.3 (35.9; 42.7)	4.7 (3.2; 6.2)
***Planned pregnancy***		*p<0.001*	*p=0.020*
Yes	1818 (44.3)	35.3 (33.1; 37.5)	5.8 (4.7; 6.9)
No	2286 (55.7)	29.8 (28.0; 31.7)	7.7 (6.6; 8.8)
***Partner support durinlg pregnancy***		*p=0.003*	*p=0.407*
A lot	3589 (88.0)	33.1 (31.6; 34.7)	6.7 (5.9; 7.6)
Little	265 (6.5)	25.3 (20.0; 30.5)	6.5 (3.5; 9.4)
None	226 (5.5)	25.7 (20.0; 31.4)	9.1 (5.3; 12.8)
***Alcohol use during pregnancy***		*p=0.957*	*p=0.433*
No	3891 (94.8)	32.3 (30.8; 33.7)	6.8 (6.0; 7.6)
Yes	212 (5.2)	32.1 (25.8; 38.4)	8.2 (4.4; 11.9)
***Type of delivery***		*p=0.006*	*p=0.064*
Vaginal	2831 (69.0)	30.9 (29.2; 32.6)	7.3 (4.4; 7.0)
C-section	1275 (31.0)	35.2 (32.6; 37.9)	5.7 (6.4; 8.3)
***Mother's SRQ at age 11 years old***		*p<0.001*	*p=0.008*
Normal	2723 (69.2)	34.7 (33.0; 36.5)	6.2 (5.2; 7.0)
Deviant	1210 (30.8)	27.2 (24.7; 29.7)	8.5 (6.9; 10.0)
***Adolescent's SDQ at age 15 years old***		*p<0.001*	*p<0.001*
Normal	2369 (60.1)	37.4 (35.5; 39.4)	4.6 (3.7; 5.5)
Borderline	518 (13.2)	26.6 (22.8; 30.5)	8.0 (5.6; 10.3)
Case	1050 (26.7)	23.1 (20.6; 35.7)	11.3 (9.4; 13.3)
***Mother's smoking during pregnancy***		*p<0.001*	*p<0.001*
No	2757 (67.2)	34.6 (32.9; 36.4)	5.5 (4.6; 6.3)
Yes	1349 (32.8)	27.3 (24.9; 29.7)	9.6 (8.1; 11.2)
***Mother's smoking during pregnancy***		*p<0.001*	*p<0.001*
No	2757 (67.2)	34.6 (32.9; 36.4)	5.5 (4.6; 6.3)
<20 cigarettes/day	1158 (28.2)	27.5 (25.0; 30.1)	9.0 (7.3; 10.6)
≥20 cigarettes/day	191 (4.6)	25.8 (19.6; 32.0)	13.8 (8.9; 18.8)
Partner's smoking during pregnancy*		*p<0.001*	*p=0.001*
No	1910 (50.5)	35.5 (27.6; 31.7)	5.5 (4.4; 6.5)
Yes	1871 (49.5)	29.7 (33.3; 37.6)	8.3 (7.0; 9.5)
**Total**	**4106**	**32.2 (30.8; 33.7**)	**6.8 (6.1; 7.6**)

The 1993 Pelotas Birth Cohort.

* Variable with the highest missing value (7.9%)

### Happiness

Happiness was defined for this analysis as a score of six points or greater on the SHS scale. Table 2 presents the association of maternal and partner smoking during pregnancy with the dichotomised SHS score. In the unadjusted analysis, there was an inverse dose response association between maternal smoking and happiness. Adolescents born to mothers who smoked 20 or more cigarettes per day during pregnancy presented a 34% reduction in probability of happiness compared to those born to non-smokers. Those born to lighter smokers had intermediate levels of risk. There was some attenuation of the risks after adjustment for confounders, and further attenuation when the mediator variable (child’s SDQ score at age 15) was also included, but the reduced risks for happiness remained statistically significant. Partner smoking was inversely associated with happiness in the unadjusted analysis and in the adjusted analysis for confounders, but not in the model adjusted for the mediator variable. 

**Table 2:** Unadjusted and adjusted analyses between smoking during pregnancy (mother and partner) and happiness.

	**Unadjusted**	Adjusted*	Adjusted**
**Variable**	**OR (95% IC)**	**OR (95% IC)**	**OR (95% IC)**
***Mother's smoking during pregnancy***	*p<0.001*	*P=0.007*	*p=0.049*
No	1.00 (ref.)	1.00 (ref.)	1.00 (ref.)
Yes	0.71 (0.61; 0.82)	0.79 (0.67; 0.92)	0.83 (0.71; 0.98)
<20 cigarettes/day	0.72 (0.62; 0.83)	0.79 (0.69; 0.93)	0.83 (0.70; 0.99)
≥20 cigarettes/day	0.66 (0.47; 0.92)	0.79 (0.55; 1.13)	0.84 (0.59; 1.22)
***Partner's smoking during pregnancy***	*p<0.001*	*p=0.046*	*p=0.087*
No	1.00 (ref.)	1.00 (ref.)	1.00 (ref.)
Yes	0.77 (0.67; 0.88)	0.86 (0.74; 1.00)	0.88 (0.76; 1.02)

The 1993 Pelotas Birth Cohort.

* Model 1: adjusted for sex, family income at birth, planned pregnancy, partner support of pregnancy, alcohol use during pregnancy, type of delivery, partner's smoking during pregnancy and mother's SRQ at age 11 years old

** Model 2: Model 1 adolescent's SDQ at age 15 years old.

### Depression

Very similar findings were observed for the adolescent depression outcome (table 3. Partner smoking was associated with depression in the unadjusted analysis only. Any maternal smoking during pregnancy was related to an 85% increase in the odds of depression in the unadjusted analysis. After adjustment for confounders, the increased odds was equal to 48%, and it was further reduced to 36% after adjustment for the mediator variable. Again, there was a dose response association with the amount of cigarettes smoked per day. The odds of depression for those born to mothers who smoked 20 or more cigarettes per day during pregnancy were 178% higher than for those born to non-smokers in unadjusted analyses. Odds ratios were reduced, but continued to be significant after adjustment for confounders and mediator. 

**Table 3:** Unadjusted and adjusted analyses between smoking during pregnancy (mother and partner) and depression.

	**Unadjusted**	Adjusted*	Adjusted**
**Variable**	**OR (95% IC)**	**OR (95% IC)**	**OR (95% IC)**
***Mother's smoking during pregnancy***	*P<0.001*	*P=0.001*	*P=0.008*
No	1.00 (ref.)	1.00 (ref.)	1.00 (ref.)
Yes	1.85 (1.45; 2.36)	1.48 (1.13; 1.94)	1.36 (1.04; 1.79)
<20 cigarettes/day	1.70 (1.31; 2;21)	1.38 (1.03; 1.84)	1.27 (0.95; 1.71)
≥20 cigarettes/day	2.78 (1.78; 4.35)	2.11 (1.31; 3.40)	1.89 (1.16; 3;08)
***Partner's smoking during pregnancy***	*P=0.001*	*P=0.150*	*P=0.157*
No	1.00 (ref.)	1.00 (ref.)	1.00 (ref.)
Yes	1.56 (1.20; 2.02)	1.23 (0.93; 1.63)	1.23 (0.92; 1.63)

The 1993 Pelotas Birth Cohort.

* Model 1: adjusted for sex, family income at birth, planned pregnancy, partner support of pregnancy, alcohol use during pregnancy, type of delivery, partner's smoking during pregnancy and mother's SRQ at age 11 years old

** Model 2: Model 1 adolescent's SDQ at age 15 years old.

### Happiness and Depression

**Figure 1:**
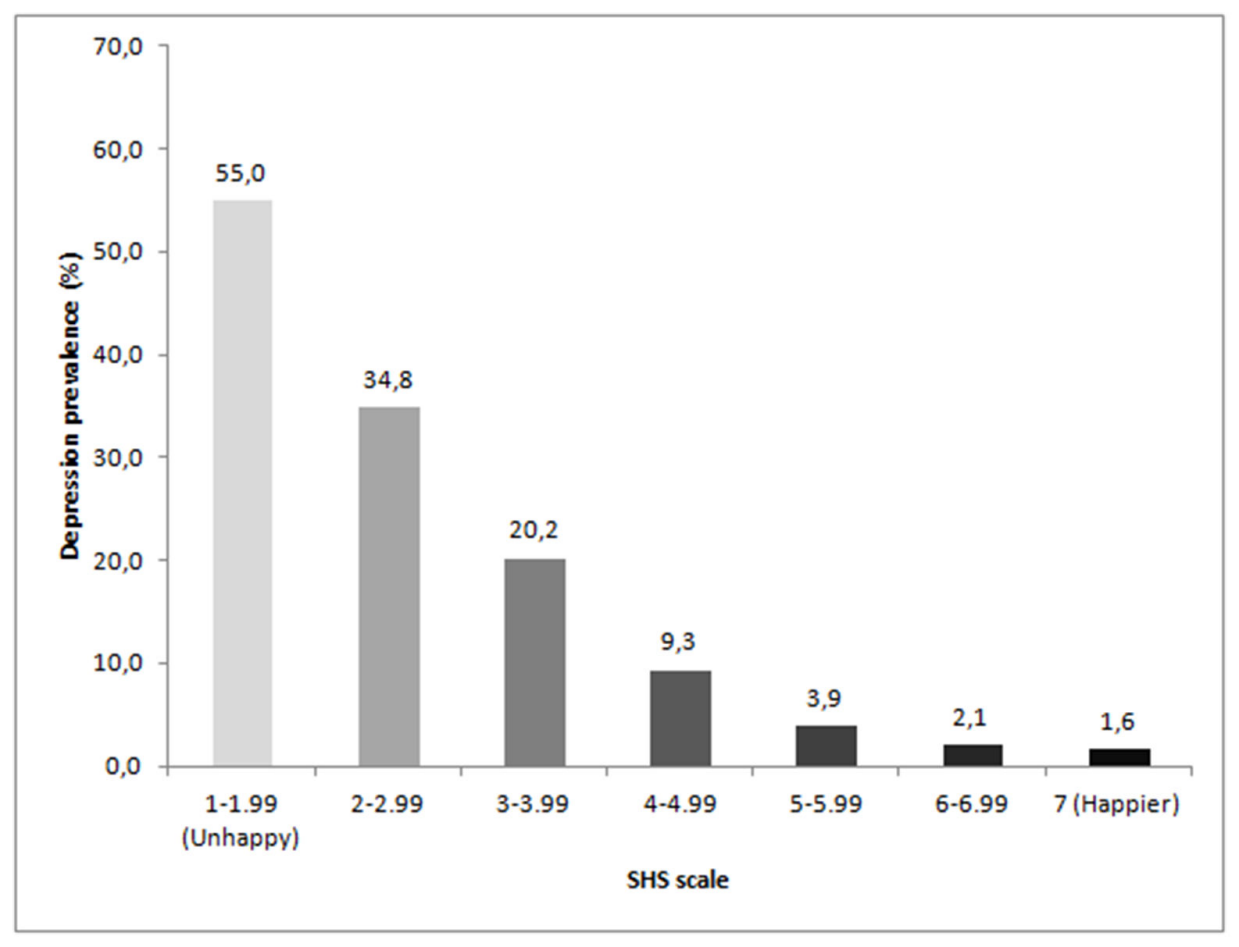
Happiness Relative to the Prevalence of Depression. The 1993 Pelotas Birth Cohort.

Figure 1 illustrates the ratings of the SHS Scale relative to the prevalence of depression. The lower the score on the SHS scale, indicating unhappy individuals, the higher the prevalence of depression (p value for trend = < 0.001). 

## Discussion

Depression and happiness do not necessarily lie on polar ends of a single spectrum and may have different underlying aetiologies. Happiness is conceptualised as an important outcome in its own right, distinct from the absence of depressive disorder. It is important to identify which predictors are common to both outcomes and which are specific to either one. Our study suggests that maternal smoking in pregnancy is important for both outcomes – increasing the risk for depression and decreasing the probability of happiness. Adolescents whose mothers and fathers had smoked during pregnancy were 21% and 14% less likely to be happy, according to the SHS scale, compared to mothers and fathers who did not smoke, after controlling for confounders. Our data also reveal a clear and robust association between prenatal maternal smoking and offspring depression at 18 years. The results remained significant after control for a range of possible confounding and mediating variables. Notably, adjustment for confounders such as maternal mental health at age 11 and mediator such as the child’s own adjustment difficulties at age 15 (total emotional, conduct, hyperactive and peer problems) did not explain the association between maternal smoking during pregnancy and offspring depression at 18. Given this, and the fact that only the mother’s smoking (not the father’s smoking) predicted offspring depression, it is “plausible” to think there is a direct biological pathway between tobacco smoke exposure in utero and depressive illness in late adolescence.

It should be highlighted that our study revealed a high prevalence of minor psychiatric disorders (SRQ) among mothers (31%) and abnormal SDQ cases among teenagers (27%). Similar rates in Brazil have been reported in other papers. A systematic review of mental disorders in the Brazilian adult population showed a prevalence ranging from 20% to 56%, affecting mainly women [31]. 

Several limitations of the present study should be noted. Women who smoke and do not quit while pregnant may be more likely to have pre-existing psychopathology such as depression, and the link with the offspring outcomes therefore might be due to pre-existing maternal mental illness and not due to smoke exposure. We did not evaluate maternal mental health during pregnancy and so could not test this hypothesis directly. However, because depression is a highly recurrent illness and the number of previous episodes increases vulnerability of further episodes, mainly among women [32,33], we adjusted our analysis for mother’s mental health at the follow-up of 11 years old as a potential confounder. The results showed that the association between smoking exposure and adolescent outcomes remained significant, even taking into account maternal mental health at age 11. We recognize that the absence of a measure of mental health during pregnancy is a limitation of our findings; the adjustment for the SRQ 11 years after pregnancy was based on the hypothesis that depression is a recurrent illness and this measure could be a proxy of previous psychiatric disorders. 

While there is no consensus in the literature about whether prenatal exposure to tobacco has a causal effect on mood and functioning, the possible biological mechanisms may involve reduced cerebrocortical binding of paroxetine (PXT), a marker for the serotonin (5HT) transporter, implicated in depression [34–36]. Rat studies suggest specific possible mechanisms. One study using rats demonstrated that prenatal nicotine exposure led to a persistent reduction of dopamine turnover in the forebrain, as well as significant reductions of serotonin turnover in the forebrain (PD22) and cerebellum (PD22) [35,37]. Nicotine is a neuroteratogen that targets synaptic function during critical developmental stages and recent rat studies indicate that Central Nervous System (CNS) vulnerability extends into adolescence. Fetal nicotine exposure in rats (gestational days 4-21) decreased the cerebrocortical binding of paroxetine (PXT), a marker for the serotonin transporter, likely indicative of a decrease in nerve terminals in that region; the effect lasted into adulthood. These results indicate that both fetal and adolescent nicotine exposure elicit apparent damage to serotonin projections with reactive increases in regions containing serotonin cell bodies [36]. 

However, studies on human beings are inconclusive about the effects on maternal smoking during pregnancy on offspring psychopathology, because the designs used in most studies (including our own) do not include measures of all factors that influence mother’s characteristics and behaviour during pregnancy, which are inherited by offspring. It is possible that the associations observed in epidemiological studies could be accounted for by genetic pathways, whereby heritable factors influence both maternal predisposition to experiencing stress, anxiety, continuation of smoking during pregnancy, and offspring outcomes. In humans, such effects are difficult to rule out without the use of experimental or genetic designs [38] and prospective epidemiological studies might not be able do address this issue [18].

Another limitation of the present study is that the use of other prenatal substances was not controlled for, for example, the evaluation of alcohol was only about consumption without further details. It is known that exposure to marijuana in utero, for example, has also been related to long-term effects [8]. Information about trimester-specific smoking could add understanding about the effects of prenatal exposure to tobacco, due to critical developmental changes occurring at specific times during gestation [39]. The information on smoking was self-reported and not validated by objective measures such as cotinine in the urine or in the blood. Recall bias is also possible for the information on partner smoking since it was investigated at delivery; the information about partner smoking was also limited due to the fact that the answer was only “yes” or “no”. Residual confounding cannot be ruled out in this study; however, the self-related information on smoking during pregnancy and about the partner probably lead to conservative results, since the harmful effects of smoking during pregnancy on the offspring are well known by the mothers.

One prior study has examined the relationship between maternal smoking in pregnancy and offspring well-being [40]. In a cohort of 7,222 children born in Copenhagen in 1959-61, and traced at 31-33 years old, those whose mothers had smoked more than 10 cigarettes a day during the last trimester of pregnancy had a significantly lower probability of high quality of adult life than those whose mothers were non-smokers (2.7% lower). This study did not include a multivariate analysis and quality of life was measured using a questionnaire that included questions related to happiness. Thus, findings from our own study about the importance of maternal smoking in pregnancy for later happiness concords with this prior examination, but it should be emphasised that “well being” is not necessarily happiness. Faden et al., studying 3 year old children of mothers who smoked during pregnancy, found that they had less well developed language, higher activity level, greater difficulty of management, fearfulness, decreased ability to get along with peers, but no effects for levels of happiness. Happiness was classified as usually happy, occasionally irritable, and irritable [41]. Prior studies have had mixed results on the effects of maternal smoking in pregnancy on offspring depression, but most were limited to childhood [6,7,42,43], and others focused on adulthood [1,44]. The only study we located with data on depression in adolescence [10] referred to ‘internalizing’ problems, including factors such as anxiety, rather than depression specifically. The study included some 2,000 children assessed at ages 11, 13 and 16 years old and almost one third of the respondents’ mothers had smoked tobacco during pregnancy. These respondents were at an increased risk for all outcomes except internalizing problems (significant odds ratios ranged from 1.40 to 2.97). Control for confounding factors reduced the strength of all relationships. In the final model, the strongest relationship was found for mothers who smoked more than 10 cigarettes a day during pregnancy and daily smoking in early adolescence (odds ratio: 1.56), but none of the relationships were statistically significant. Our own finding that depression at age 18 years is strongly linked to maternal smoking in pregnancy contrasts with results from this single other study that examined the issue. This difference in results may be at least in part due to the isolation of depression as an outcome in our own study rather than a combination of outcomes described as internalizing problems. 

Although our findings showed that offspring were less likely to be happy if their mothers or fathers smoked during pregnancy, the information about smoking by the father was referred by the mother, and it was not known the amount of cigarettes smoked by the father; therefore we believe that the most reliable results from this study are those about smoking during pregnancy by the mother. 

 Given the inability of human research to adequately account for all environmental and genetic selection factors, we must be cautious about conclusions of a “causal effect” from maternal pregnancy smoke on offspring outcomes. Our findings showed an “association” between an increase risk for depression and a decrease in the probability of happiness for offspring whose mothers smoked during pregnancy, even when adjusted for potential confounders and a possible mediator, but we can not conclude that there is a “causal pathway” in this association due to the design of the study.
